# Oral and Fecal Microbiome in Molar-Incisor Pattern Periodontitis

**DOI:** 10.3389/fcimb.2020.583761

**Published:** 2020-10-08

**Authors:** Pâmela Pontes Penas Amado, Dione Kawamoto, Emmanuel Albuquerque-Souza, Diego Castillo Franco, Luciana Saraiva, Renato Corrêa Viana Casarin, Anna Carolina Ratto Tempestini Horliana, Marcia Pinto Alves Mayer

**Affiliations:** ^1^Department of Microbiology, Institute of Biomedical Sciences, University of São Paulo, São Paulo, Brazil; ^2^Division of Periodontology, Department of Stomatology, School of Dentistry, University of São Paulo, São Paulo, Brazil; ^3^Department of Biological Oceanography, Oceanographic Institute, University of São Paulo, São Paulo, Brazil; ^4^Institute of Environmental Sciences, Faculty of Biology, Jagiellonian University, Kraków, Poland; ^5^Department of Prosthodontics and Periodontics, Piracicaba Dental School, State University of Campinas, São Paulo, Brazil; ^6^Biophotonics Applied to Health Sciences, University Nove de Julho, São Paulo, Brazil

**Keywords:** dental plaque, aggressive periodontitis, oral microbiome, fecal microbiome, human microbiome, 16S rRNA sequencing, *Aggregatibacter actinomycetemcomitans*, dysbiosis

## Abstract

In order to improve our understanding on the microbial complexity associated with Grade C/molar-incisor pattern periodontitis (GC/MIP), we surveyed the oral and fecal microbiomes of GC/MIP and compared to non-affected individuals (Control). Seven Afro-descendants with GC/MIP and seven age/race/gender-matched controls were evaluated. Biofilms from supra/subgingival sites (OB) and feces were collected and submitted to *16S rRNA* sequencing. *Aggregatibacter actinomycetemcomitans* (*Aa*) JP2 clone genotyping and salivary nitrite levels were determined. Supragingival biofilm of GC/MIP presented greater abundance of opportunistic bacteria. *Selenomonas* was increased in subgingival healthy sites of GC/MIP compared to Control. *Synergistetes* and *Spirochaetae* were more abundant whereas *Actinobacteria* was reduced in OB of GC/MIP compared to controls. *Aa* abundance was 50 times higher in periodontal sites with PD≥ 4 mm of GC/MIP than in controls. GC/MIP oral microbiome was characterized by a reduction in commensals such as *Kingella, Granulicatella, Haemophilus, Bergeyella*, and *Streptococcus* and enrichment in periodontopathogens, especially *Aa* and sulfate reducing *Deltaproteobacteria*. The oral microbiome of the *Aa* JP2-like+ patient was phylogenetically distant from other GC/MIP individuals. GC/MIP presented a higher abundance of sulfidogenic bacteria in the feces, such as *Desulfovibrio fairfieldensis, Erysipelothrix tonsillarum*, and *Peptostreptococcus anaerobius* than controls. These preliminary data show that the dysbiosis of the microbiome in Afro-descendants with GC/MIP was not restricted to affected sites, but was also observed in supragingival and subgingival healthy sites, as well as in the feces. The understanding on differences of the microbiome between healthy and GC/MIP patients will help in developing strategies to improve and monitor periodontal treatment.

## Introduction

Periodontitis is a multifactorial inflammatory disease that affects periodontal tissues in response to a dysbiotic microbial community (Hajishengallis, 2015). The most common forms of periodontitis observed in clinical practice were recently included in a single large category (Caton et al., 2018; Papapanou et al., 2018), and the previous localized aggressive periodontitis is now classified as molar/incisor pattern periodontitis (MIP) (Tonetti et al., 2018). Although MIP shares some general features with other forms of periodontitis, its classification as a distinct disease entity is still under discussion (Fine et al., 2018, 2019).

MIP, as the name suggests, affects incisors and first molars of adolescents and young adults associated to minimal plaque and rapid rate of progression (Fine et al., 2019). Genetic characteristics and high incidence of MIP in members of the same family support a strong familial aggregation (Meng et al., 2007). Despite its rare prevalence worldwide (0.1–2%), adolescents of African and Middle Eastern descent present a 10-fold higher risk for MIP than other populations (Fine et al., 2019). MIP is commonly associated with the putative pathogen *Aggregatibacter actinomycetemcomitans* (*Aa*) (Slots et al., 1980; Zambon et al., 1983), and the highly virulent strains belonging to the JP2 clone are associated with disease progression in young Afro-descendants (Bueno et al., 1998; Haubek et al., 2008; Höglund Åberg et al., 2014; Ennibi et al., 2019).

Currently, it is well-known that periodontitis is induced by the activity of the entire microbial community in the subgingival biofilm of affected sites, characterized by higher amounts of pathogens, and decreased proportion of commensal microorganisms (Faveri et al., 2009; van Essche et al., 2013) and not by a single pathogen (Fine et al., 2013a). However, there are no reports on the composition of complex bacteria communities associated with MIP using next generation sequencing methods.

Moreover, periodontitis has been associated to several systemic diseases and inflammatory disorders of the gastrointestinal tract (Kumar, 2017), although a closer look at this two-way relationship is still necessary. Hypothetically, the dysbiotic oral microbiota not only induces a periodontal hyperinflammatory response, but it would be a source of persistent systemic inflammation, serving as inoculum for the gut. Indeed, the oral administration of *Aa* and *Porphyromonas gingivalis* in mice causes alterations in the fecal microbiota (Arimatsu et al., 2014; Komazaki et al., 2017). In addition, the fecal microbiome of individuals affected by more common forms of periodontitis showed increased levels of *Firmicutes, Proteobacteria, Verrucomicrobia* and *Euryarchaeota*, and decreased levels of *Bacteroidetes* compared to healthy individuals (Lourenςo et al., 2018). However, a link between the oral and the fecal microbiomes in patients with unusual periodontitis forms such as MIP is still not established.

In the present study, we tested the hypothesis that patients with MIP present a dysbiotic microbiome, which would reflect not only on the subgingival microbiota of affected sites, but also on other oral niches such as the biofilms of supragingival and subgingival healthy sites, and even on the gut.

## Materials and Methods

### Cross-Sectional Study Individuals

Afro-descendants with periodontitis Stage III, Grade C/MIP (GC/MIP), aged between 18 and 25 years, and age/race/gender-matched non-affected individuals (Control) were selected at the School of Dentistry of the University of São Paulo (FOUSP), after protocol approval by Research Ethics Committee at Biomedical Sciences Institute of University of São Paulo (number: 1.821.309). Those who agreed to participate in the study signed an informed consent form.

GC/MIP cases were diagnosed as follows: interdental clinical attachment loss (CAL) ≥5 mm at the site of greatest loss; radiographic alveolar bone loss extending at least to middle third of the root; % bone loss/age >1.0; early onset disease (18–25 years), angular alveolar bone defects, and MIP (Tonetti et al., 2018). The inclusion criteria for the Control were as follows: lack of sites with PD >3 mm (assuming no pseudo pockets); bleeding on probing (BoP) ≤ 20% (Joss et al., 1994); no caries or extensive restoration; and at least 28 permanent teeth.

Exclusion criteria were previous subgingival periodontal therapy (scaling and root planning and/or periodontal surgical therapy); use of medications that could affect the periodontium, such as corticosteroids or antibiotic treatment in the previous 6 months and/or mouthwashes containing antimicrobials; systemic diseases that could affect the progression of periodontitis (e.g., diabetes and immunological disorders); pregnant or lactating; and smokers.

### Clinical Assessment

BoP was evaluated based on the presence (1) or absence (0) of bleeding up to 30 s after probing; probing depth (PD) measured as the distance (in millimeters) from the free gingival margin to the bottom of the pocket; gingival recession (GR) measured as the distance from the cementum-enamel junction to the free gingival margin; CAL was measured as PD plus GR. When there was no GR, CAL was determined as the distance from the cementum-enamel junction to the bottom of the pocket. All parameters were obtained at six sites per tooth (mesiobuccal, buccal, distobuccal, distolingual, lingual and mesiolingual), except for third molars using a North Carolina probe (Hu-Friedy, Chicago, USA). Mean and standard deviation for PD and CAL were calculated by full-mouth, as well as number of sites with PD ≥4 mm and PD ≥6 mm. Measurements reproducibility was calculated by intra-class correlation coefficient for PD (ICC = 0.86) and CAL (ICC = 0.85) in two separate examinations.

### Samples Collection

All biofilm samples were collected from one site per quadrant in the molar-incisor region using sterile periodontal curettes and were pooled according to the location (supra or subgingival) and periodontal probing depth ( ≤ 3 or ≥4 mm) in Tris-EDTA buffer. Supragingival (SpA_GC/MIP) and subgingival (SbA_GC/MIP) biofilms samples were collected from sites with the highest pocket depths (PD ≥4 mm) of GC/MIP. Biofilms from healthy subgingival sites (PD ≤ 3 mm and lack of BoP) were collected from GC/MIP (SbH_GC/MIP). Supragingival (SpH_Control) and subgingival (SbH_Control) biofilms were collected from control individuals.

Fecal samples were self-collected using a sterilized recipient, stored at −20°C, and transported in ice. Unstimulated saliva was collected by passively drooling into a chilled tube for 5 min. Saliva samples were centrifuged at 14,000 × g for 20 min at 4°C (Siqueira et al., 2012) and the supernatant was kept. All samples were stored at −80°C.

### Genotyping of *Aggregatibacter actinomycetemcomitans*

Quantitative PCR was used to detect the *Aa* JP2-like+ clone in oral samples of GC/MIP and Control. Primers and a probe to detect the *orfX*′ region were used (Yoshida et al., 2012). The reactions were performed in triplicate as follows: 1 μL of DNA (pooled supra and subgingival biofilm samples of each individual), 0.1 μL of probe, 300 nM of each primer and 10 μL of TaqMan Master Mix (Applied Biosystem, Foster City, CA, USA) to a total 20 μL. The amplification cycle was 50°C/2′, 95°C/10′, followed by 40 cycles of 95°C/15′′ and 60°C/1′ in the thermocycler Step One Plus Real-Time PCR System (Applied Biosystem, Foster City, CA, USA).

### Biofilm Samples Processing and Sequencing

DNA was extracted using the MasterPure DNA Purification Kit (Epicentre Biotechnologies, Madison, WI, USA) for oral samples and the QIAamp® DNA Stool Mini Kit (Qiagen, Hilden, Germany) for fecal samples, following the manufacturer's protocol. DNA quality was determined using Qubit 2.0 fluorometer (Thermo-Fisher Scientific, Carlsbad, CA, USA).

A barcoded primer set based on universal primers Bakt_341F CCTACGGGNGGCWGCAG and Bakt_805R GACTACHVGGGTATCTAATCC (Herlemann et al., 2011) was used to amplify the hypervariable V4–V5 region of *16S rRNA* gene. DNA samples were sequenced (Macrogen, Seoul, Republic of Korea) using the Illumina MiSeq 2 × 250 platform, following the manufacturer's protocol. Illumina sequences data were submitted to Sequence Read Archive (SRA) under BioProject identification number PRJNA580506.

### Sequencing Data Analyses

Raw sequencing pair-end reads were assembled using PEAR software v0.9.10 (Zhang et al., 2014), with a minimum overlap of 20 bp and with an e-value cutoff of 4e-10. Reads were filtered for length (≥440 bp), quality score (mean >30) using USEARCH v10.0.240_i86linux32 (Edgar, 2010), and sequences were clustered into operational taxonomic units (OTU) at 97% similarity. Reads were filtered for bacterial sequences for further analyses in QIIME 1.8.0 pipeline (Caporaso et al., 2010). Taxonomy was assigned using the Human Oral Microbiome Database (HOMD) v.15.1. Bacteria unclassified and unknown were collapsed and named as “others” to generate relative abundance plots. Alpha diversity was determined by Chao 1, ACE, Shannon, and Simpson indexes and the amount of unique OTUs estimated. OTU tables of each pair groups were normalized using cumulative sum scaling (CSS). Beta diversity was evaluated considering Weighted UniFrac distances and visualized by Principal Coordinates Analysis (PCoA). Core microbiomes consisting of species detected in 50% of the samples from each site of GC/MIP and Control were obtained using QIIME. Oral biofilm (OB) refers to the sum of reads of each taxon detected in all oral biofilms samples per group: SpA+SbH+SbA of GC/MIP and SpH+SbH of Control. OB and feces exclusive and shared species were identified using Venny v.2.1.

### Levels of Nitrite in Saliva

Saliva nitrite levels were evaluated in GC/MIP and Control samples in three independent assays, as described in a previous study (Han et al., 2013). Fifty microliter of unstimulated saliva supernatants were mixed with equal volumes of Griess reagent in triplicate in 96 wells plates. After 10 min, the optical density at 540 nm was determined and compared to a standard curve consisting of sodium nitrate in PBS (pH 7.2) at different concentrations.

### Statistical Analysis

GC/MIP individuals were selected among all patients referred to the Periodontology clinics at FOUSP who met the inclusion/exclusion criteria over the recruitment period of 2 years. Sample size was estimated according to a previous study analyzing multiple parameters (Shaddox et al., 2016), which required a minimum of 6 GC/MIP cases to reach a power of 80%. Differences in clinical parameters and salivary nitrite levels between groups were calculated based on the Mann-Whitney test in BioEstat® software v5.3. Non-parametric *t*-test using Monte Carlo simulation was applied to compare relative abundance data in QIIME. Student *t*-test was applied to compare alpha diversity data between groups. Permutational Multivariate Analysis of Variance (PERMANOVA) (vegan::adonis) was used to quantify beta diversity differences between groups. Spearman's Rank test was applied to determine correlations levels between clinical parameters, nitrite levels, and relative abundance of species using RStudio® v3.4.4. The significance level of all tests was set at 5% (*p* < 0.05).

## Results

### Demographic/Periodontal Parameters

This case control-study consisted of 7 GC/MIP individuals and 7 age-gender-race-matched controls. Only Afro-descendants were included in view of MIP demographic specificities. No gender restrictions were used during the recruitment period, but only women appropriately followed the inclusion/exclusion criteria. GC/MIP presented higher percentage of BoP, and higher PD and CAL mean compared to Controls (*p* < 0.01; Table 1).

**Table 1:** Demographic/periodontal clinical parameters and *A. actinomycetemcomitans* (*Aa*) prevalence and abundance in the oral microbiome.

**Variables**	**GC/MIP**	**Control**
**Age**	21.29 ± 2.29	21.29 ± 2.29
**BoP**	43.54 ± 16.58^*^	15.00 ± 5.05
**PD**		
Full mouth	2.25 ± 0.65^*^	1.71 ± 0.31
Sites with PD ≥4 mm	4.8 ± 0.8	ND
Sites with PD ≥6 mm	6.7 ± 0.7	ND
**CAL**		
Full mouth	2.85 ± 0.3^*^	ND
Sites with PD ≥4 mm	4.9 ± 1.0	ND
Sites with PD ≥6 mm	6.9 ± 0.6	ND
***Aa*** **prevalence**		
Supragingival biofilm	7/7	7/7
Subgingival biofilm (PD ≤ 3 mm)	7/7	7/7
Subgingival biofilm (PD ≥4 mm)	7/7	ND
***Aa*** **relative abundance**		
Supragingival biofilm	0.09%	0.05%
Subgingival biofilm (PD ≤ 3 mm)	0.14%	0.06%
Subgingival biofilm (PD ≥4 mm)	2.92%^$^	ND
**Detection of** ***Aa*** **JP2-like clone**	1^§^/7	0/7

*The groups consisted of 7 individuals with GC/MIP and 7 age-gender-race-matched controls. Probing depth (PD) and clinical attachment loss (CAL) values are represented as mean ± standard deviation in sites with PD ≥4 and ≥6 mm. Bleeding on probing (BoP) is represented by percentage mean ± standard deviation. Subgingival biofilm samples of sites with PD ≤ 3 mm correspond to healthy sites, while subgingival biofilm samples of sites with PD ≥4 mm correspond to affected sites. Mann-Whitney test was used to evaluate inter-group differences in clinical parameters and Non-parametric t-test to evaluate relative abundance differences. The significance level of the tests was set at 5% (p <0.05)*.

* *Mann-Whitney test, p <0.05*.

$ *Non-parametric t-test, p <0.05; Aa relative abundance in the subgingival biofilm of sites with PD ≥4 mm of GC/MIP was compared to its abundance in the subgingival biofilm of sites with PD ≤ 3 mm of Control*.

§ *Only 1 GC/MIP patient was Aa JP2-like+ by qPCR analysis. ND, not determined*.

### Bacterial Community Profiling

The oral and fecal microbiomes were determined by *16S rRNA* sequencing of a total of 48 samples, which generated 3,396,028 high quality paired-end reads (average of 70,751 reads per sample). A total of 3,315 OTUs were distributed among 12 phyla, 79 families and 551 species.

### Taxonomical Analyses Revealed That the Dysbiosis Was Not Only a Characteristic of Affected Sites

Taxa abundance in supra and subgingival biofilms were compared between GC/MIP and Control. Since subgingival affected sites are only present in GC/MIP, data on SbA_GC/MIP were compared to SbH_Control.

**Figure 1:**
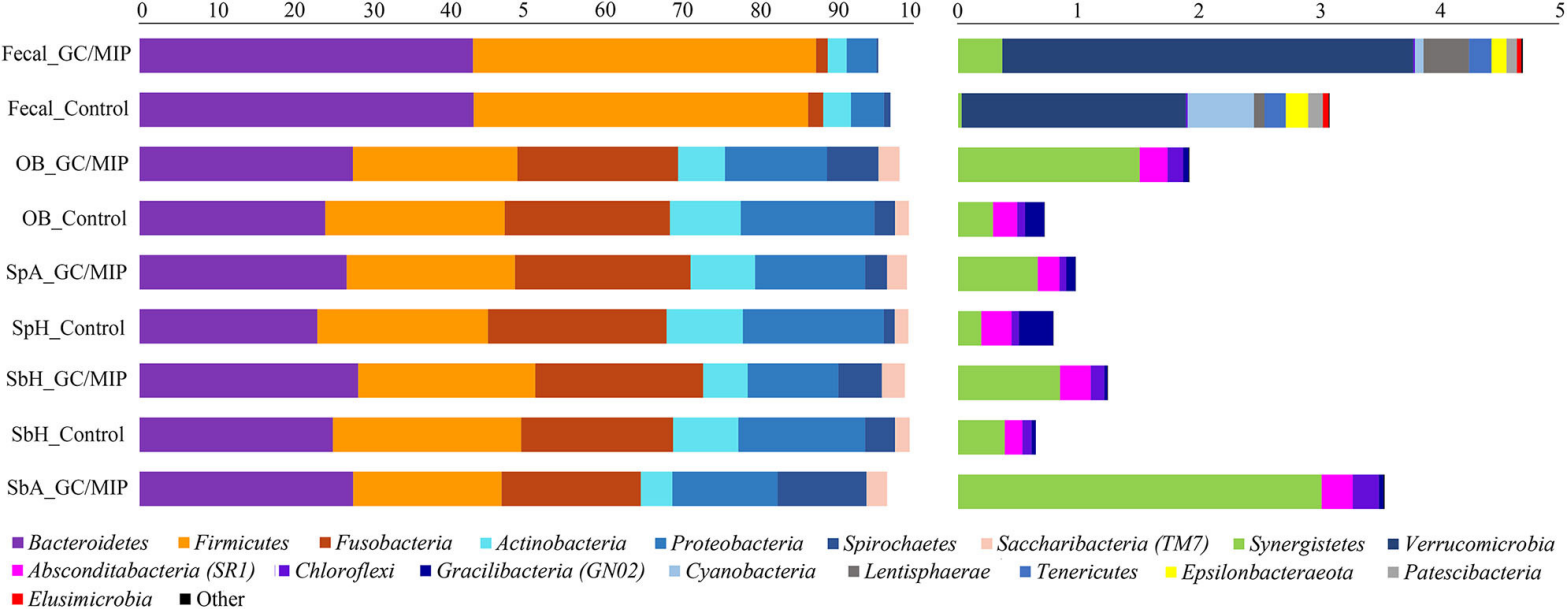
Phyla relative abundance (%) based plots. The left plot represents the most abundant phyla (>1%) and the right plot represents the least abundant phyla (<1%) detected in GC/MIP and Control samples: fecal samples (Fecal_GC/MIP and Fecal_Control), oral biofilm samples (OB_GC/MIP and OB_Control, where OB refers to the sum of reads of each taxon detected in all biofilm samples per group: SpA+SbH+SbA in GC/MIP and SpH+SbH in Control groups), supragingival biofilms (SpA_GC/MIP and SpH_Control), subgingival healthy sites (SbH_GC/MIP and SbH_Control), and subgingival affected sites (SbA_GC/MIP).

**Figure 2:**
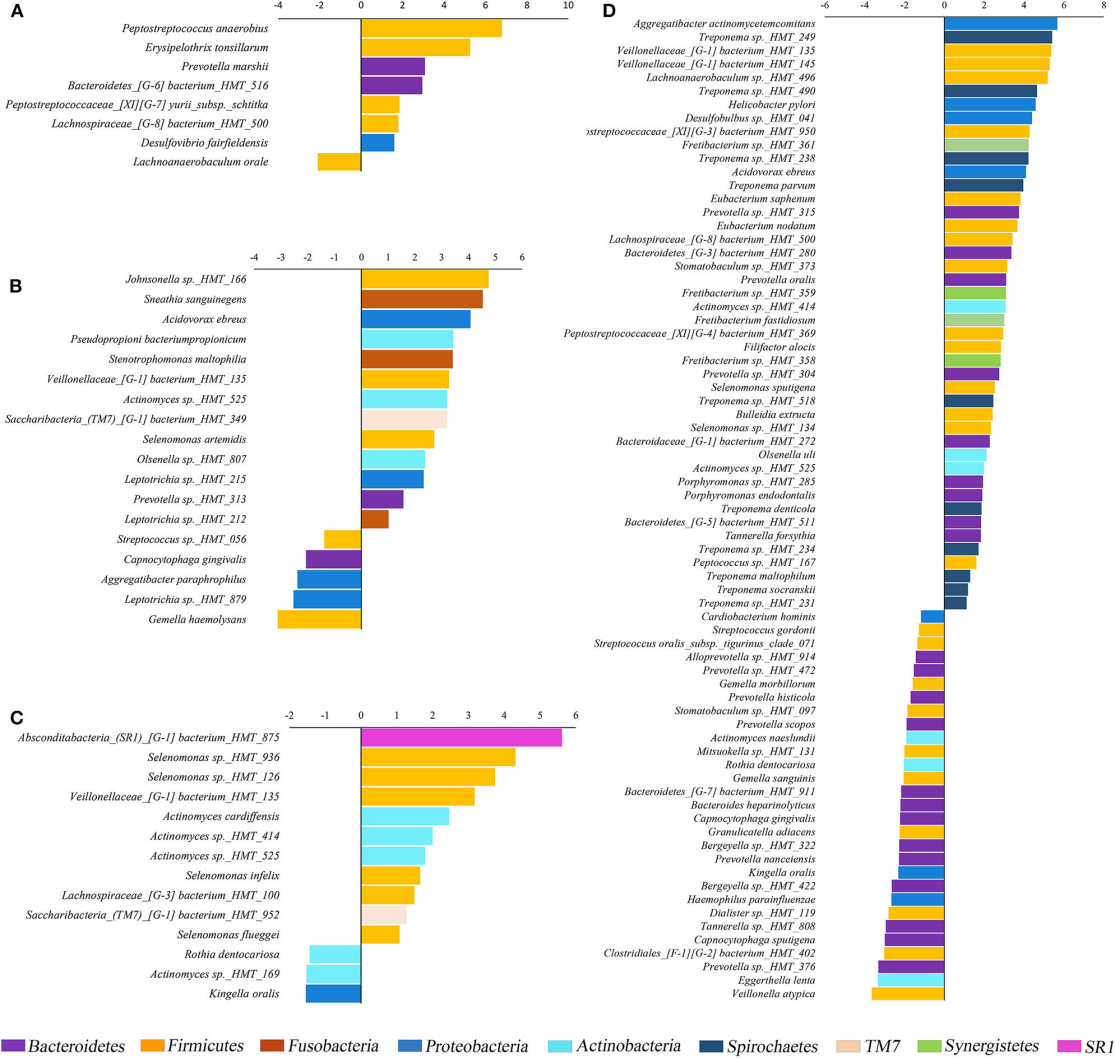
Fold changes (log_2_) of species relative abundance. Species detected in lower (negative values) or higher abundance (positive values) in GC/MIP samples compared to Control in: fecal samples **(A)**, supragingival sites **(B)**, subgingival healthy sites **(C)**, and subgingival affected sites of GC/MIP compared to subgingival healthy sites of Control **(D)**. Only species that significantly differed between groups were displayed (*p* < 0.05, Non-parametric *t*-test).

**Figure 3:**
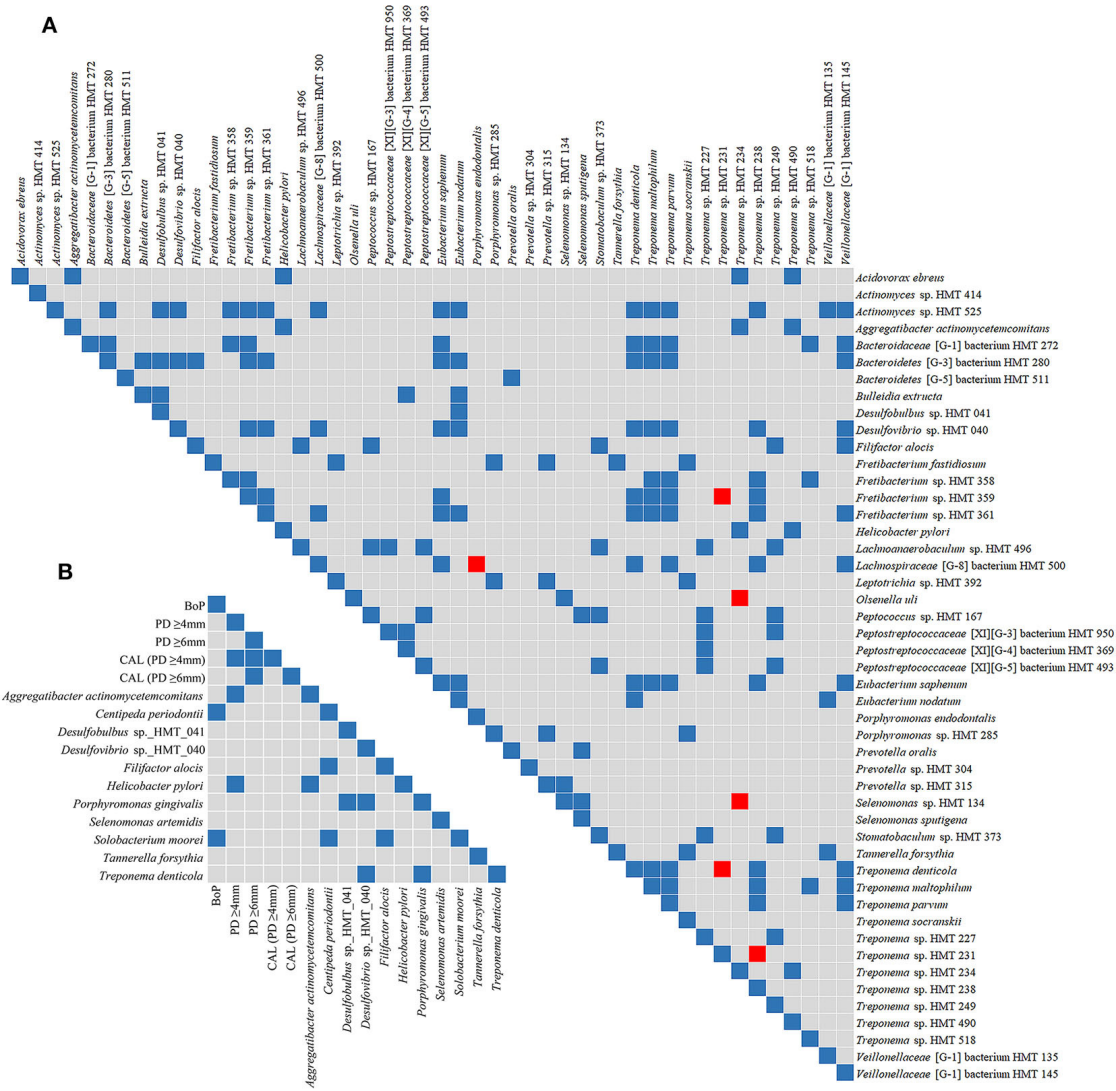
Spearman's rank correlation matrices. Matrix **(A)** corresponds to the correlation between species that were more abundant in subgingival affected sites of GC/MIP (SbA_GC/MIP) when compared to subgingival sites of healthy Control. Matrix **(B)** corresponds to the correlation between the abundance of sulfidogenic bacteria in SbA_GC/MIP and periodontal clinical parameters. Blue squares indicate positive correlations (*R* ≥ 0.75, *p* < 0.05) and red squares indicate negative correlations (R ≤ −0.75, *p* < 0.05).

**Figure 4:**
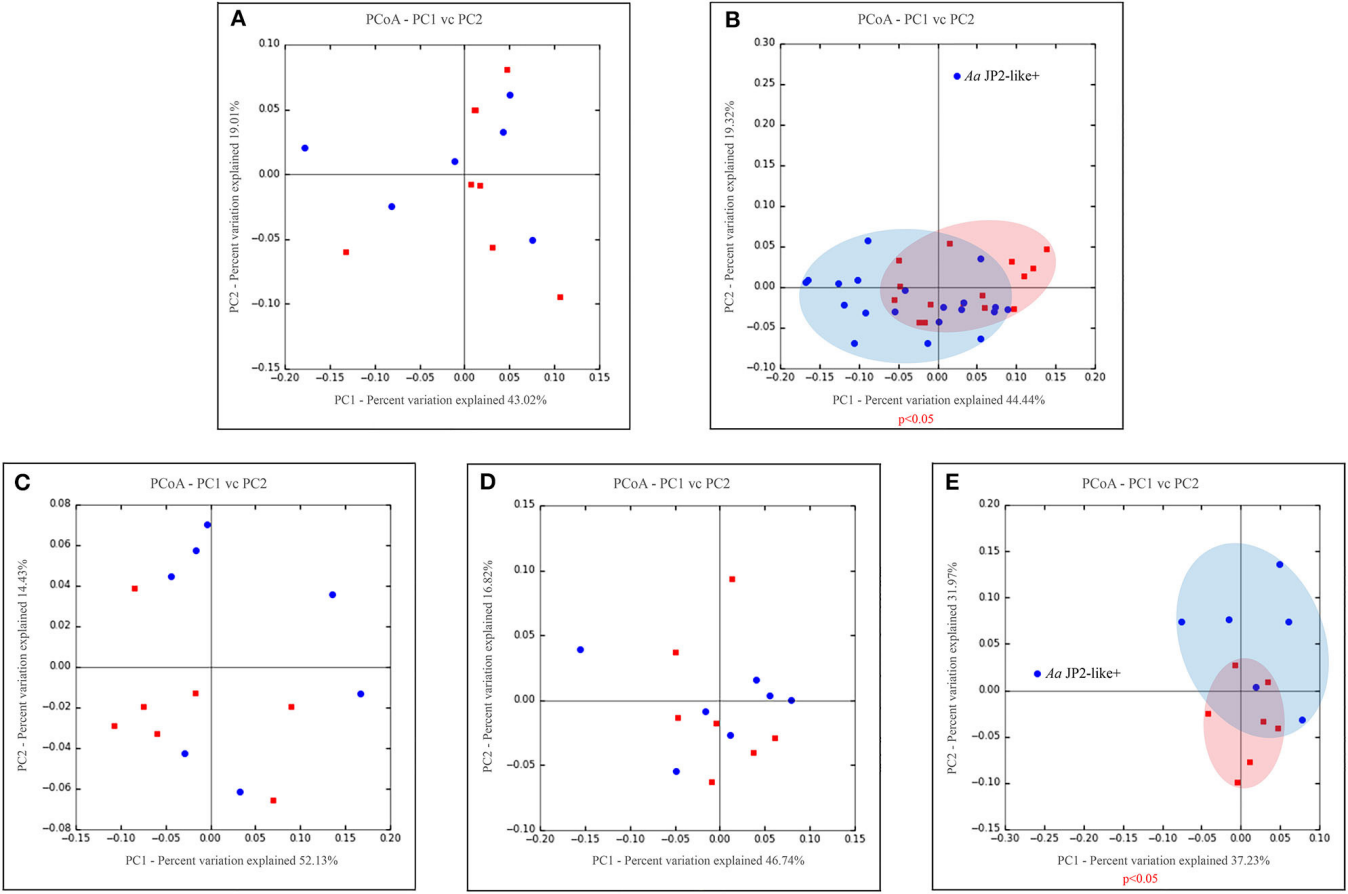
Principal coordinate analysis based on weighted UniFrac distance metric. Graphics represents beta diversity analysis between samples: fecal **(A)**, oral (supra + subgingival sites sampled) **(B)**, supragingival sites **(C)**, subgingival non-affected sites of GC/MIP and Control and **(D)**, subgingival affected sites of GC/MIP and subgingival sites of Control **(E)**. Blue dots correspond to GC/MIP samples and red dots correspond to Control samples. Blue or light red ellipses indicate groups of samples closely related. A significance level of 5% was applied by using PERMANOVA test (vegan::adonis). Only one GC/MIP case harbored the *Aa* JP2-like+ clone, which clustered apart from the other GC/MIP samples, as signalized in **(B,E)**.

Phyla detected in all samples are shown in Figure 1. *Bacteroidetes, Firmicutes, Fusobacteria*, and *Proteobacteria* were predominant in oral samples. *Synergistetes* and *Spirochaetae* were more abundant in OB_GC/MIP and in SbA_GC/MIP, while increased levels of *Actinobacteria* were found in OB_Control (*p* < 0.05). Differences in species abundance in the fecal microbiome between groups are shown in Figure 2A. Differences in species abundance between GC/MIP and Control in supragingival biofilm and subgingival healthy sites were observed (Figures 2B,C). The most impressive differences were detected when subgingival affected sites of GC/MIP (SbA_GC/MIP) were compared to SbH_Control (Figure 2D). Although *Aa* was detected in all oral samples (Table 1), it was 50 times more abundant in SbA_GC/MIP than in SbH_Control (log2FC = 5.6). Moreover, *Aa* abundance in SbA_GC/MIP positively correlated to the abundance of *Acidovorax ebreus* (*R* = 0.97/*p* = 0.0003)*, Helicobacter pylori* (*R* = 0.99/*p* = 2.93e-06), *Treponema* sp._HMT_234 (*R* = 0.86/*p* = 0.01), and *Treponema* sp._HMT_490 (*R* = 0.98/*p* = 7.962e-05; Figure 3A). When comparing the periodontal clinical parameters to the abundance of sulfidogenic bacteria in SbA_GC/MIP, sites with PD ≥4 mm positively correlated to the abundance of *Aa* (*R* = 0.82/*p* = 0.02) and *H. pylori* (*R* = 0.81/*p* = 0.03), while BoP positively correlated to the abundance of *Centipeda periodontii* (*R* = 0.84/*p* = 0.02) and *Solobacterium moorei* (*R* = 0.81/*p* = 0.03; Figure 3B). Supplementary Table 1 shows the relative abundance of species in oral biofilms, which differed between GC/MIP and Control. Alpha diversity of oral and fecal samples did not differ between groups (Supplementary Figure 1). Beta diversity analysis revealed that OB and SbA_GC/MIP samples tended to cluster apart from OB and SbH_Control samples, respectively (*p* < 0.05; Figure 4).

### The Oral Microbiome of the *Aa*-JP2-like+ Patient Was Distinct From the Other Individuals

Considering that, all individuals harbored *Aa* in oral samples, and its higher abundance in OB of GC/MIP compared to controls (Table 1), we evaluated the presence of the highly virulent *Aa* JP2-like+ clone in subgingival biofilm samples. Samples from all individuals were tested, however *Aa*-JP2-like+ clone was detected in only one GC/MIP individual (Table 1). The OB microbiome of this individual (GC/MIP 1) clustered apart from all OB and subgingival samples (Figures 4B,E). The abundance of *Aa* in biofilm samples of GC/MIP 1 was 18.8 times higher than observed in Controls, and 5.8 times higher than the remaining GC/MIP individuals.

**Figure 5:**
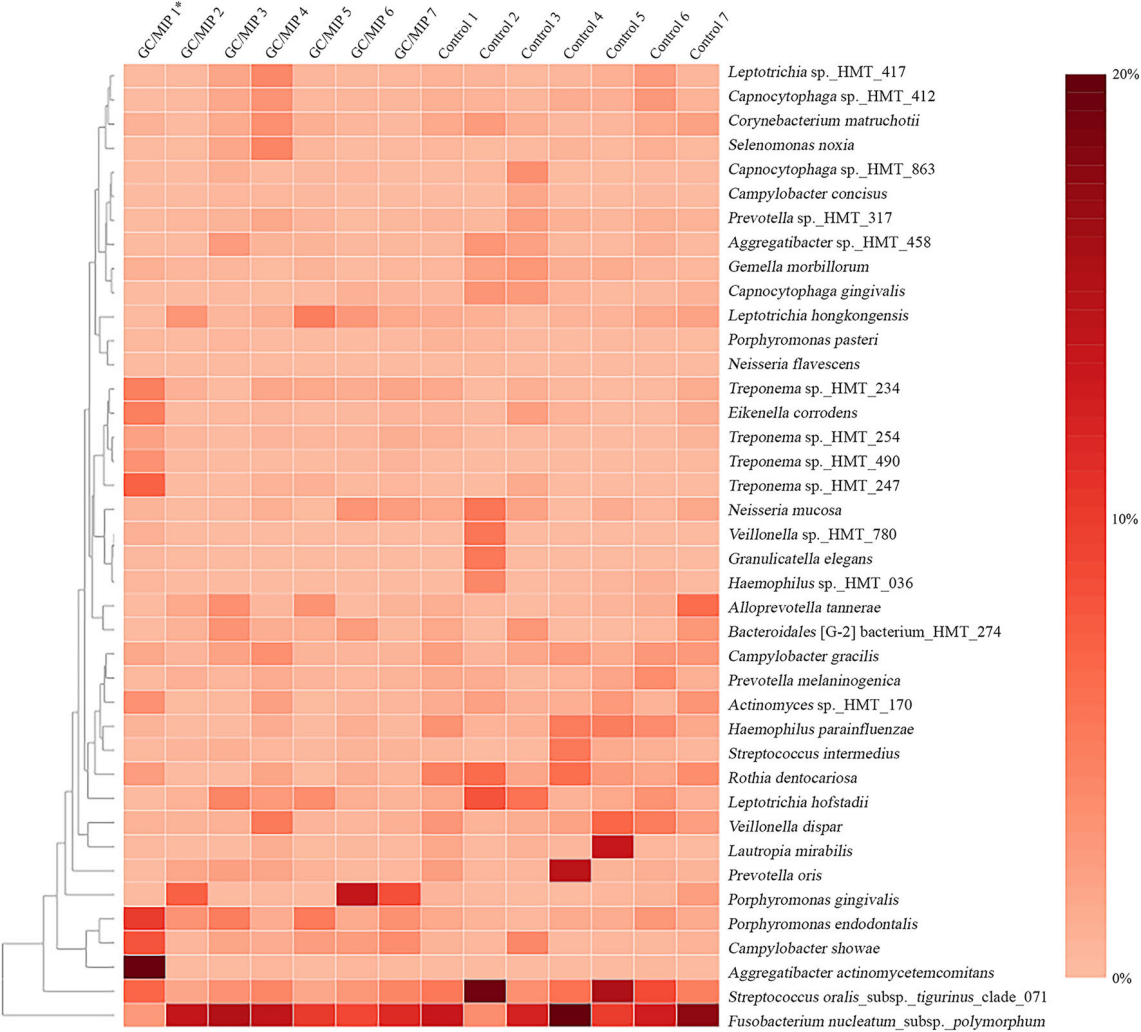
Heat map based on the relative abundance of the 40 most abundant species in the subgingival biofilm of individuals with GC/MIP and Control. Species relative abundance of subgingival affected sites of GC/MIP (SbA_GC/MIP) was compared to subgingival sites of Control (SbH_Control). *Patient harboring *Aa* JP2-like+ clone.

Differences in patterns of microbial communities between the *Aa*-JP2-like+ patient and the other studied individuals were evaluated by plotting a heat map based on the relative abundance of the 40 most abundant species in subgingival sites (Figure 5). Clusters formed by *Aa, Campylobacter showae* and *Porphyromonas endodontalis*, and by *Treponema* sp._HMT_247, 490, 254, 234, and *Eikenella corrodens* were detected in the *Aa*-JP2-like+ subgingival affected sites.

### Dysbiosis in the Fecal Microbiome

*Bacteroidetes* and *Firmicutes* were predominant in fecal samples (Figure 1). We highlight the increased abundance of sulfate-reducing bacteria (SRB) *Peptostreptococcus anaerobius, Erysipelothrix tonsillarum*, and *Desulfovibrio fairfieldensis*, and reduced abundance of Lachnoanaerobaculum orale in fecal samples of GC/MIP compared to Controls (Figure 2A). The fecal microbiome of the *Aa*-JP2-like+ patient did not differ from the other GC/MIP individuals (Supplementary Figure 2).

### Core Microbiome

**Figure 6:**
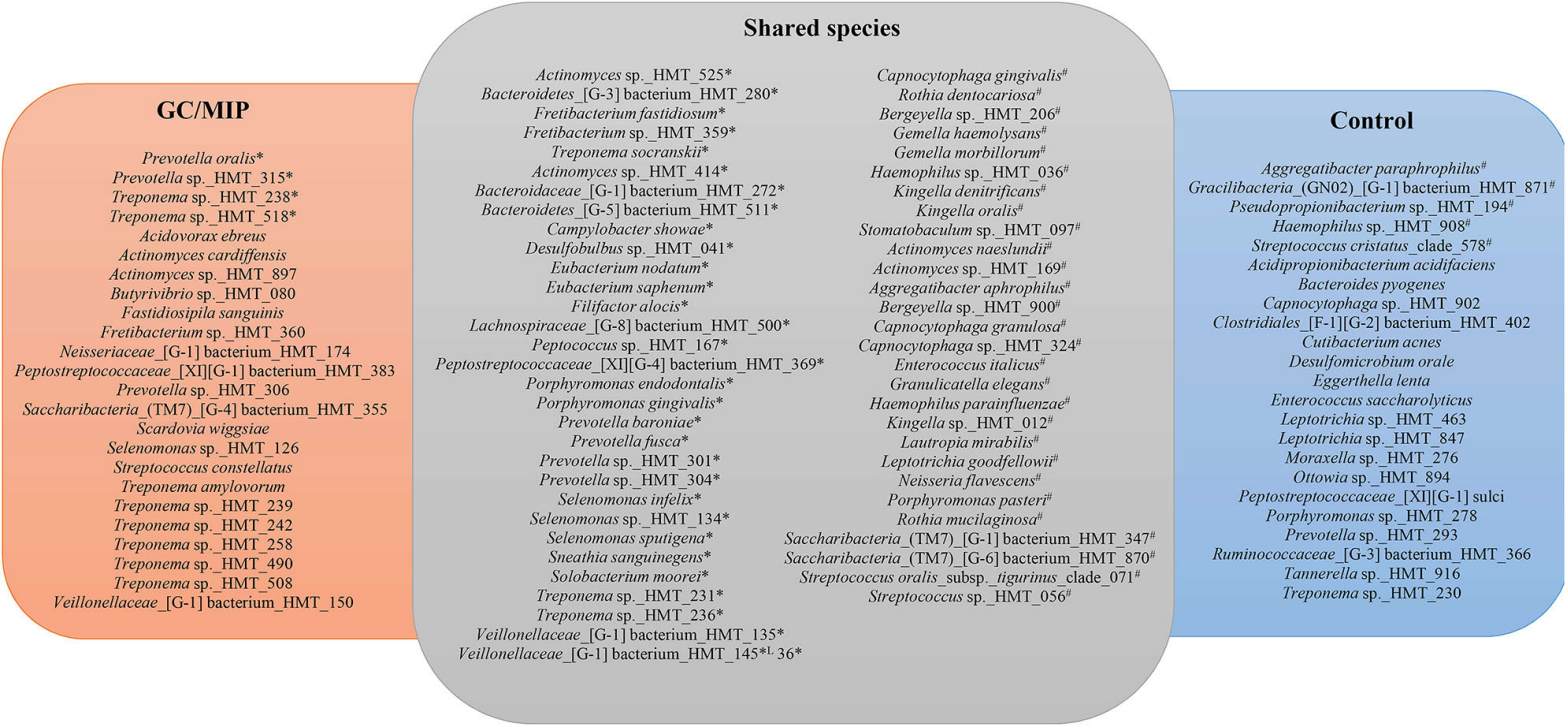
Core microbiome based on species present in the oral biofilm of 50% of the individuals of GC/MIP, Control and shared species. Oral biofilm refers to the sum of reads of each taxon detected in all oral biofilm samples per group: (SpA+SbH+SbA) in GC/MIP and (SpH+SbH) in Control groups. *Species more abundant in GC/MIP. ^#^Species more abundant in Control (*p* < 0.05, Non-parametric *t*-test).

Core microbiome analysis revealed shared and exclusive taxa of at least 50% GC/MIP and Control individuals. The oral core microbiome revealed a total of 291 species shared by both groups, while 24 were exclusively detected in GC/MIP and 23 in Control. Due to the large number of shared species in the oral biofilm, only those that were statistically different between groups are shown in Figure 6. The complete list is in Supplementary Table 2. The fecal core microbiome revealed that 251 species were shared by both groups, while 21 species were exclusively detected in GC/MIP and 35 in Control individuals (Supplementary Table 3).

### Nitrite Levels in GC/MIP

Since the abundance of some nitrate-reducing bacteria (NRB) differed in OB from GC/MIP and controls, nitrite levels in saliva were determined and correlated with oral bacteria composition and periodontal clinical parameters. Despite the higher amount of salivary nitrite in GC/MIP compared to Control, this difference was not significant (Supplementary Figure 3). However, nitrite levels positively correlated with the number of sites with PD ≥4 mm (*R* = 0.77/*p* = 0.04), with the percentage of sites with CAL ≥6 mm (*R* = 0.75/*p* = 0.05), and with the abundance of *Fretibacterium fastidiosum* (*R* = 0.91/*p* = 0.004) and *Treponema socranskii* (*R* = 0.89/*p* = 0.007) in SbA_GC/MIP.

## Discussion

The present study revealed new aspects of MIP and contributed to the understanding of dysbiotic microbiome involved in the etiopathogenesis of this phenotype of periodontitis at the oral and intestinal levels. To the best of our knowledge, this is the first study evaluating the microbiome of GC/MIP, and not only subgingival sites were evaluated but also supragingival biofilms and fecal samples.

Localized aggressive periodontitis is quite rare in Brazil, Europe, and North America, but there is a need to understand this disease. Due to the low prevalence of GC/MIP, we recruited any patient diagnosed with this condition for 2-years throughout the study, and matched health individuals in relation to age, gender, and ethnical background. Only subjects of African descent were selected due to the association of MIP and the JP2 clone with race (Haubek, 2010; Fine et al., 2019). However, even with this strategy, the prevalence of the JP2 clone, known to be originated in Africa (Haubek, 2010), was very low, differing from studies in African Americans and Moroccans (Burgess et al., 2017; Ennibi et al., 2019). All subjects were under 26 years of age, since aging leads to changes in oral microbial communities (Claesson et al., 2017). These strict selection criteria resulted in a more homogenous group, but limited the number of subjects. Furthermore, there is still a need to highlight differences among microbial communities of the different phenotypes of periodontitis. However, comparison of MIP with the highly prevalent slow progressing periodontitis, known as chronic periodontitis (CP), would impose age differences, adding another variable to the analysis, due to the phenomena of inflammaging (Franceschi et al., 2007).

The supragingival biofilms of GC/MIP were characterized by a greater abundance of opportunistic bacteria when compared to control, such as *Sneathia sanguinegens, Stenotrophomonas maltophilia, Selenomonas artemidis*, and *Johnsonella* sp._HMT_166, which were recently associated to periodontitis (Pérez-Chaparro et al., 2018). Differences were also observed when subgingival healthy sites of GC/MIP were compared to Control since SbH_GC/MIP microbiome was characterized by higher abundance of *Actinomyces* and *Selenomonas* species. Interestingly, *S. sputigena*, found in greater abundance in SbA_GC/MIP than in controls, is considered a periodontopathogen due to its virulence factors and was suggested as a marker of disease activity (Hiranmayi et al., 2017). Altogether, these data indicated that GC/MIP presents altered microbial communities in supragingival biofilm, as well as in subgingival healthy sites. We also reinforced the association between *Aa* and GC/MIP cases due to its expressive greater abundance in affected sites. However, despite the association of the JP2 clone with MIP in Afro-descendants, only one GC/MIP individual harbored the more virulent JP2 clone indicating the virulence potential of the other *Aa* strains. The promoter region of the *lkt* operon differs in the JP2 clone when compared to other *Aa* strains, leading to the production of high amounts of leukotoxin (Brogan et al., 1994). However, certain non-JP2 strains may also produce high amounts of leukotoxin (Höglund Åberg et al., 2014) and differences between the JP2 clone and non-JP2 strains do not rely only in the production of the leukotoxin (Huang et al., 2013). Furthermore, the virulence potential of *Aa* strains vary in relation to other virulence factors such as cagE, a putative exotoxin (Johansson et al., 2017), and cytolethal distending toxin (Fabris et al., 2002).

The distinct microbial composition in periodontitis sites of the *Aa*-JP2-like+ MIP patient indicated its potential to alter the periodontal environment and, consequentially, the bacterial community. Inflammation is known to modulate the resident microbiota, and *Aa* activates the inflammasome in macrophages, leading to secretion of IL-1β (Ando-Suguimoto et al., 2020). The highly leukotoxic JP2 clone may play a role as a key stone pathogen and alter the response promoted by the microbial community, possibly leading to an altered oral microbiome in JP2 carriers. The higher amount of leukotoxin may affect interaction with immune cells, due to its ability to decrease phagocytosis and killing, but also to induce the release of metalloproteases and to activate the inflammasome (Johansson, 2011), possibly contributing to periodontitis progression. However, we evaluated only one JP2+ subject, and this hypothesis should be tested in larger populations.

The abundances of eleven *Treponema* species were increased in SbA_GC/MIP, including putative periodontopathogens such as *T. denticola, T. maltophilum, T. parvum*, and *T. socranskii*, some of which were previously associated with GC/MIP (Faveri et al., 2009; Fine et al., 2013b). Eight species of *Treponema* were part of the oral core microbiome. Moreover, a positive correlation between *Aa, Treponema* sp._HMT_234 and 490 was observed, and abundance of *Treponema* positively correlated with abundance of *Fretibacterium* in SbA_GC/MIP. Colonization levels of *F. fastidiosum* and the *Synergistetes* phylotypes HMT_358, 359 and 361 were increased in SbA_GC/MIP compared to SbH_Control. To date, *Synergistetes* cluster A, which includes *F. fastidiosum*, was increased in the saliva of individuals affected by generalized periodontitis (Belibasakis et al., 2013), and this specie is considered putative periodontopathogen (Oliveira et al., 2016; Deng et al., 2017).

On the other hand, we observed a reduction of health-associated species in all oral sites of GC/MIP when compared to Controls. Abundance of 29 species was lower in SbA_GC/MIP than in SbH_Control, including *Kingella oralis, Granulicatella adiacens*, and *Haemophilus parainfluenzae* and species of the genera *Bergeyella, Capnocytophaga, Gemella, Prevotella*, and *Streptococcus*. Thus, the biofilm with high levels of initial colonizers and beneficial bacteria was found in controls, whereas in MIP, the microbiota switched toward higher levels of strictly anaerobic bacteria even in supra (SpA_GC/MIP) and subgingival biofilms of healthy sites (SbH_GC/MIP). These data indicated that the dysbiosis observed in MIP is not only associated with deep pockets, but is also found in supragingival plaque and subgingival healthy sites. Previous data had also reported differences in the microbial communities when subgingival sites from control were compared to shallow pockets in CP patients, suggesting that the shallow pockets in persons with disease may represent an intermediate stage in disease development (Griffen et al., 2012). Thus, treatment strategies of MIP should aim not only the reduction of pathogens, but also favor beneficial bacteria both in supra and subgingival sites.

Abundance of sulfidogenic bacteria (H_2_S-producers in anaerobic conditions), such as *Aa, Porphyromonas gingivalis, Treponema denticola, Tannerella forsythia, Filifactor alocis, Centipeda periodontii, Selenomonas artemidis, Solobacterium moorei*, and *Helicobacter pylori*, as well as *Deltaproteobacteria*, increased in the oral microbiome of GC/MIP. The *Deltaproteobacteria Desulfobulbus* sp._HMT_041 and *Desulfovibrio* sp._HMT_040 were more abundant in SbA_GC/MIP than in SbH_Control. The role of anaerobic SRB in periodontitis has been suggested (Campbell et al., 2013). Furthermore, an increased abundance of *Desulfobulbus* sp._HMT_041 was observed in subgingival sites of GC/MIP adolescents prior to disease development (Fine et al., 2013b) and this phylotype was associated with generalized and refractory periodontitis (Colombo et al., 2009; Oliveira et al., 2016). The affected sites with PD ≥4 mm presented a higher abundance of *Aa* and *H. pylori*. Disease severity and higher PD were previously associated with *H. pylori* in patients with periodontitis (Dye et al., 2002; Hu et al., 2016). The possible mechanisms involved in disease severity caused by *H. pylori* may be linked to alterations in the subgingival microbiota and inflammation by inducing the production of IL-6, IL-8, and INF-γ (Hu et al., 2016). Although several evidences associate *H. pylori* to periodontitis, especially to CP, its contribution to disease development and/or progression is still unclear (Liu et al., 2020).

Hydrogen Sulfide (H_2_S) can lead to generation of nitric oxide (NO) and vice-versa (Grossi, 2009; Tran et al., 2014), suggesting a synergistic production of these toxic metabolites in periodontitis. Since the microbiome approach can only indicate the identity of the microorganisms present at a specific site, whereas their functions could only be inferred, we determined nitrite levels in saliva samples. Salivary nitrite levels were higher in GC/MIP patients than in controls, although this difference was not statistically significant possibly due to a diluted effect promoted by the localized disease pattern. However, salivary nitrite levels positively correlated with abundance of sulfidogenic bacteria such as *Treponema socranskii*. These findings raised the hypothesis that the production of NO and H_2_S may contribute to the shift from a balanced to a dysbiotic microbiota in MIP, favoring and being favored by the periodontal destruction. In this line, NRB such as *Acidovorax ebreus* were more abundant in GC/MIP oral samples than in controls, and their role in the etiopathogenesis of periodontitis deserves further investigation.

The fecal microbiome of GC/MIP also presented a higher abundance of sulfidogenic bacteria, such as *Desulfovibrio fairfieldensis, Erysipelothrix tonsillarum*, and *Peptostreptococcus anaerobius*, when compared to controls and the last two species were part of the fecal core microbiome of GC/MIP. *D. fairfieldensis* and *P. anaerobius* can be detected in the gastrointestinal tract of healthy individuals, and their oral colonization was previously associated to periodontitis (Loubinoux et al., 2002; Colombo et al., 2016). Furthermore, *D. fairfieldensis* is associated with serious infections and abscesses in the gut (Pimentel and Chan, 2007). *P. anaerobius*, in its turn, was enriched in fecal samples and biopsies from patients with colorectal cancer (CRC), and induced colon dysplasia in a CRC animal model (Tsoi et al., 2017). On the other hand, as far as we know, *E. tonsillarum* has not been associated with any oral or systemic diseases in humans. The fecal microbiome features observed in GC/MIP differed from other findings in patients with CP (Lourenςo et al., 2018), supporting the hypothesis that MIP should be considered a distinct periodontal disease (Fine et al., 2019).

A high abundance of SRB was reported in colonic biopsies of Afro-Americans but not in other ethnical groups (Yazici et al., 2017). Moreover, SRB is a potential risk factor for CRC development in Afro-Americans. Intriguingly, the association between CRC and periodontitis has been suggested (Lauritano et al., 2017). The patients included in our study did not report any other health problem, suggesting that studies are needed to elucidate the oral–intestinal network in MIP, and the possible role of oral bacteria in gut inflammatory conditions. We have recently shown that MIP is characterized by an altered profile of chemokines in saliva (Kawamoto et al., 2020), and this altered response may play a role in modulating the microbiome of both mucosa surfaces. Since MIP diagnosis can be done early in life, a prospective long-term evaluation should insert periodontitis into a multiprofessional health care approach, and should evaluate the role of the altered immune response in MIP in modulating the resident microbiota and vice-versa.

In summary, these preliminary data shed light on the microbiome associated to GC/MIP cases in Afro-descendants, indicating that dysbiosis occurs not only in subgingival affected sites, but also in supragingival biofilms and healthy subgingival sites, as well as in the fecal microbiome. GC/MIP oral microbiome was characterized by high levels of known putative periodontopathogens such as *Aa, Treponema* and *Selenomonas* species, but also by a decreased abundance of biofilm early colonizers and beneficial bacteria. In addition, less recognized putative pathogens such as H_2_S-producing bacteria, including *Deltaproteobacteria* and NRB such as *Acidovorax ebreus* may play an important role in MIP and should be further investigated. The more integrated view of oral and intestinal microbiomes described in this study is a glimpse on the comprehension of the etiopathogenesis of complex and severe forms of periodontitis such as GC/MIP.

## Data Availability Statement

The datasets presented in this study can be found in online repositories. The names of the repository/repositories and accession number(s) can be found at: https://www.ncbi.nlm.nih.gov/bioproject/PRJNA580506/.

## Ethics Statement

The studies involving human participants were reviewed and approved by Research Ethics Committee at Biomedical Sciences Institute of University of São Paulo (number: 1.821.309). The patients/participants provided their written informed consent to participate in this study.

## Author Contributions

PA contributed to conception, design, data acquisition, analysis and interpretation, and drafted the manuscript. DK and LS contributed to conception, design, and data acquisition. EA-S contributed to interpretation and drafted the manuscript. DF contributed to data analysis. RC contributed to data acquisition and analysis. AH contributed to data acquisition. MM contributed to conception, design, data acquisition and interpretation, and drafted the manuscript. All authors critically revised the manuscript and gave final approval. The authors agree to be accountable for all aspects of the work.

## Conflict of Interest

The authors declare that the research was conducted in the absence of any commercial or financial relationships that could be construed as a potential conflict of interest.
