# Supplementary material for: Oral and Fecal Microbiome in Molar-Incisor Pattern Periodontitis
Source: Front Cell Infect Microbiol. 2020 Oct 8;10:583761. doi: 10.3389/fcimb.2020.583761 (PMC7578221; doi:10.3389/fcimb.2020.583761)
Supplement: Supplementary file 4 [file Data_Sheet_1.docx]

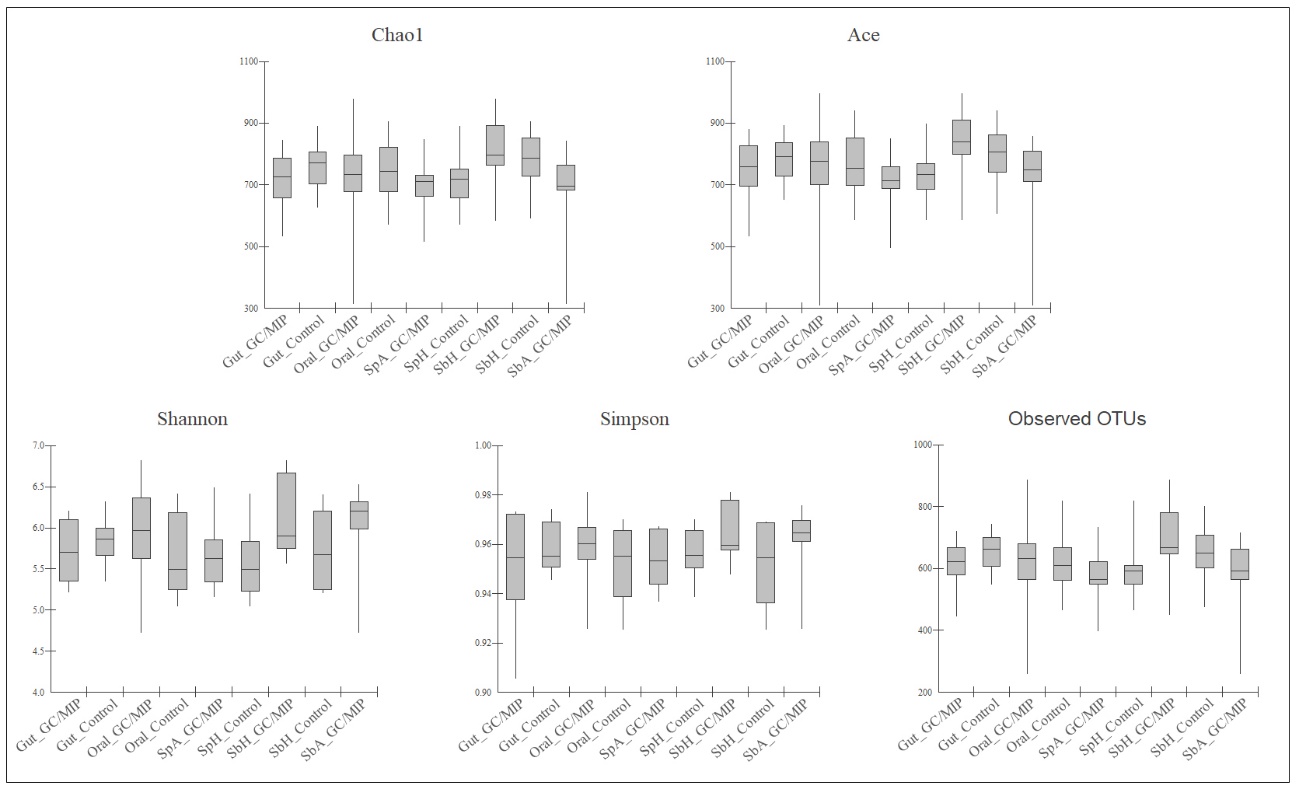


**Supplementary Figure 1.** Box-plots of alpha diversity indexes calculated for each site. Alpha diversity was determined by the indexes Chao 1 (estimates richness), ACE (Abundance-based Coverage Estimator), Shannon (estimates diversity and evenness), Simpson (estimates evenness) and the amount of unique OTUs found in each sample was estimated (observed species). Boxes contain 50% of all values and whiskers represent the 25th and 75th percentiles. No statistically significant difference was observed (*P* > 0.05, Student *t* test).
