# Supplementary material for: Oral and Fecal Microbiome in Molar-Incisor Pattern Periodontitis
Source: Front Cell Infect Microbiol. 2020 Oct 8;10:583761. doi: 10.3389/fcimb.2020.583761 (PMC7578221; doi:10.3389/fcimb.2020.583761)
Supplement: Supplementary file 5 [file Data_Sheet_2.docx]

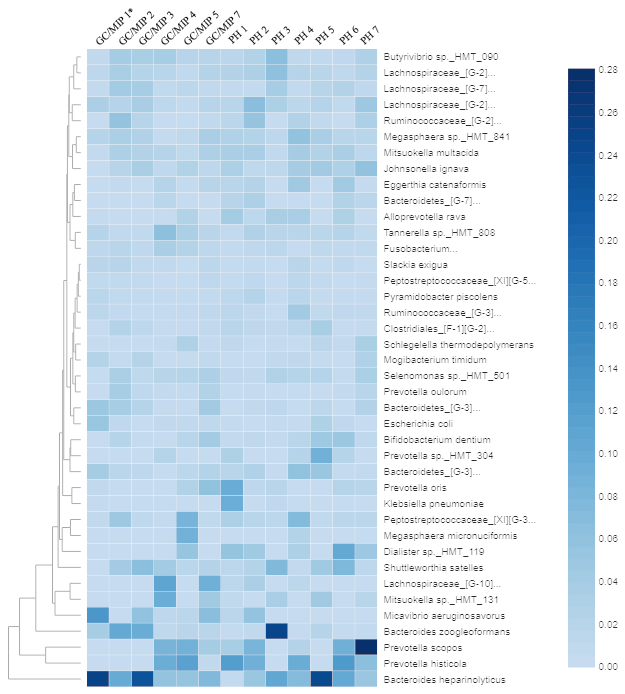


**Supplementary Figure 2.** Heat map based on the relative abundance of the 40 most abundant species in fecal samples of individuals with GC/MIP and Control. *, patient *Aa* JP2-like+ clone.
