# Supplementary material for: Oral and Fecal Microbiome in Molar-Incisor Pattern Periodontitis
Source: Front Cell Infect Microbiol. 2020 Oct 8;10:583761. doi: 10.3389/fcimb.2020.583761 (PMC7578221; doi:10.3389/fcimb.2020.583761)
Supplement: Supplementary file 6 [file Data_Sheet_3.docx]

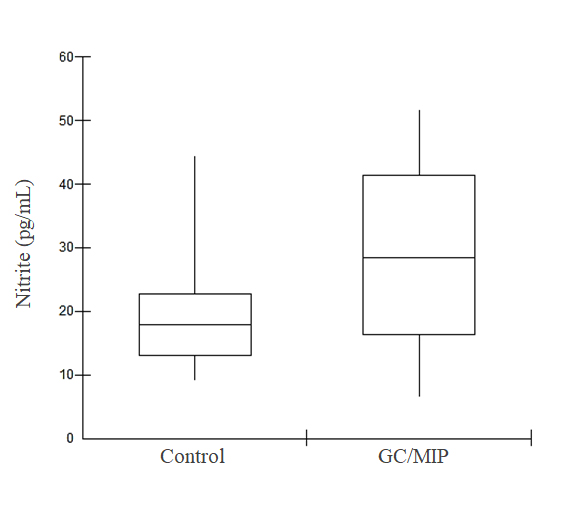


**Supplementary Figure 3.** Box-plots of nitrite concentrations in the stimulated saliva supernatant of GP/MIP and Control individuals. Boxes contain 50% of all values and whiskers represent the 25th and 75th percentiles. No statistically significant difference was observed (*P* > 0.05, Mann-Whitney test).
